# Niches and Seasonal Changes, Rather Than Transgenic Events, Affect the Microbial Community of *Populus* × *euramericana* ‘Neva’

**DOI:** 10.3389/fmicb.2021.805261

**Published:** 2022-01-28

**Authors:** Yali Huang, Yan Dong, Yachao Ren, Shijie Wang, Yongtan Li, Kejiu Du, Xin Lin, Minsheng Yang

**Affiliations:** ^1^Institute of Forest Biotechnology, Forestry College, Hebei Agricultural University, Baoding, China; ^2^Hebei Key Laboratory for Tree Genetic Resources and Forest Protection, Baoding, China; ^3^Agricultural Office of Kenfeng Subdistrict Office, Tangshan, China

**Keywords:** *Populus* × *euramericana* ‘Neva’, 16S rRNA, ITS2, genetic transformation, microbial community

## Abstract

Exploring the complex spatiotemporal changes and colonization mechanism of microbial communities will enable microbial communities to be better used to serve agricultural and ecological operations. In addition, evaluating the impact of transgenic plants on endogenous microbial communities is necessary for their commercial application. In this study, microbial communities of *Populus* × *euramericana* ‘Neva’ carrying *Cry1A*c-Cry3A-BADH genes (ECAA1 line), *Populus* × *euramericana* ‘Neva’ carrying *Cry1Ac-Cry3A-NTHK1* genes (ECAB1 line), and non-transgenic *Populus* × *euramericana* ‘Neva’ from rhizosphere soil, roots, and phloem collected in different seasons were compared and analyzed. Our analyses indicate that the richness and diversity of bacterial communities were higher in the three *Populus* × *euramericana* ‘Neva’ habitats than in those of fungi. Bacterial and fungal genetic-distance-clustering results were similar; rhizosphere soil clustered in one category, with roots and phloem in another. The diversity and evenness values of the microbial community were: rhizosphere soil > phloem > root system. The bacterial communities in the three habitats were dominated by the Proteobacteria, and fungal communities were dominated by the Ascomycota. The community composition and abundance of each part were quite different; those of *Populus × euramericana* ‘Neva’ were similar among seasons, but community abundance fluctuated. Seasonal fluctuation in the bacterial community was greatest in rhizosphere soil, while that of the fungal community was greatest in phloem. The transgenic lines ECAA1 and ECAB1 had a bacterial and fungal community composition similar to that of the control samples, with no significant differences in community structure or diversity among the lines. The abundances of operational taxonomic units (OTUs) were low, and differed significantly among the lines. These differences did not affect the functioning of the whole specific community. Sampling time and location were the main driving factors of changes in the *Populus* × *euramericana* ‘Neva’ microbial community. Transgenic events did not affect the *Populus* × *euramericana* ‘Neva’ rhizosphere or endophytic microbial communities. This study provides a reference for the safety evaluation of transgenic plants and the internal colonization mechanism of microorganisms in plants.

## Introduction

Microorganisms play an important role in soils and many eukaryotic host organisms. Each plant species hosts a genotype-specific core microbiome, dynamically responding to environmental factors. Rhizosphere soil is the most important area for interactions between plants and soil, and the growth of rhizosphere microorganisms depends on exudates released from roots that stimulate their growth (Pandit et al., 2020; Moisan et al., 2021). “Endophyte” is a generic term for any organism that lives inside of a plant, which interacts with the plant host as mutualists or commensals at some point of their life cycle (Wilson, 1995) without changing plant morphology, and ubiquitously found in all plant species and probably co-evolved with higher plants (Krings et al., 2007). Endophytic microorganisms also play an important role in plant growth, nutrient accumulation, enhancement of stress tolerance, and pest control (Hardoim et al., 2008; Ownley et al., 2010; Hallmann et al., 2011). Any changes in the endophytic community diversity of plants, or their activity, may have a significant impact on plant growth and environmental adaptability (Gaiero et al., 2013; Vandenkoornhuyse et al., 2015), but we know relatively little at present about the distribution and diversity of endophytes in many plant groups and plant communities.

Poplar is one of the most important economic tree species in the world. Because of its rapid growth, strong adaptability, high yield, short rotation period, and wide use, the poplar industry has developed rapidly, which has benefited China’s economic development and ecologically sound construction. With the increase in planting area of single poplar species, serious problems with insect pests have greatly restricted the development of the poplar industry. In addition, China contains 99.13 million hectares of saline-alkali land, there are currently few poplar varieties suitable for growth in arid and saline-alkali areas. The cultivation of insect-resistant and salt-tolerant poplar varieties is of great value to the cultivation of poplar ecological forests (Sun et al., 2010). Applying modern molecular biology technology, i.e., transferring multiple resistance genes into poplar, can substantially shorten the breeding process. The *Cry1Ac* and *Cry3A* insecticidal protein genes have toxic effects on Lepidoptera and Coleoptera pests, respectively, while *BADH* and *NTHK1* are two important salt-tolerant genes. Both insecticidal protein genes and salt-alkali resistance genes have been transferred into poplar, which has resulted in great economic value and ecologically significant improvements in the resistance of poplar.

While genetic transformation brings great social benefits, environmental safety is a topic of concern. Microorganisms are sensitive to changes in external conditions and can respond to soil degradation or improvement. The aboveground litter, root exudates, and root residues of transgenic plants all carry exogenous gene products that may directly affect soil microbial populations and lead to changes in plant–microbe interactions (Bruinsma et al., 2003; Lynch et al., 2005). Study on the effects of genetic transformation have focused mainly on transgenic crops and microbial communities in rhizosphere soil, and there has been less research on the effects of transgenic trees on such soil, particularly the endophytic microbial flora component (Guan et al., 2016). Since the root systems of trees are in contact with the soil for a long time, the effects of transgenic trees on soil and endogenous microorganisms are more noteworthy than those of crops. The development of next-generation sequencing (NGS) technology has led to studies of the microbial communities of transgenic plants. In this study, *Populus* × *euramericana* ‘Neva’ (*PEN*) carrying *Cry1Ac-Cry3A-BADH* genes and *PEN* carrying *Cry1Ac-Cry3A-NTHK1* genes grown in the field were used as research materials, and the non-transgenic *PEN* was used as a control. The composition and diversity of the bacterial and fungal communities in the rhizosphere soil, roots, and phloem of the two transgenic lines and the control in different seasons were monitored, and the temporal and spatial variations in microbial communities were explored and compared. The results provide a theoretical basis for research on the safety of the transgenic poplar soil ecosystem and the poplar microbial community colonization mechanism.

## Materials and Methods

### Site Description and Sampling

*PEN* is a new fast-growing female poplar that is widely used in commercial production. Three lines of *PEN* carrying *Cry1Ac-Cry3A-BADH* genes (ECAA1–ECAA3) and three lines carrying *Cry1Ac-Cry3A-NTHK1* genes (ECAB1–ECAB3) were obtained as follows. The *Cry1Ac* and *Cry3A* genes were linked together and constructed into the same vector with the *BADH* and *NTHK1* genes, respectively, producing clones of *PEN* by agrobacterium-mediated transformation (Liu et al., 2016; Yang et al., 2016). Resistant roots of the transformed plants were detected through polymerase chain reaction (PCR), fluorescence quantitative PCR, and enzyme-linked immunosorbent assay (ELISA) analyses of the toxic protein. Indoor and field insect-feeding experiments were conducted, growth and morphological observations were made, and insect-resistant characteristics were noted. The six lines of *PEN* have been approved by the State Forestry and Grassland Bureau for environmental release.

In April, 2018, a comparative experimental forest was planted in Mancheng District, Baoding City, Hebei Province (E115°28′ and N38°52′). Using a random block design, three replicates were established, with nine plants per plot, a plant spacing of 2 m, row spacing of 4 m, and a total area of 6666 m^2^. Natural conditions and management practices were consistent across the test site. In June, August, and October, 2020, rhizosphere soil, root, and phloem samples of the highly resistant lines ECAA1 (hereafter, A1) and ECAB1 (B1) were collected, and *PEN* was used as a control. One plant was randomly selected from each plot, three replicates, i.e., nine lines were collected each time. After the litter layer on the ground had been removed, soil was excavated to a depth of about 30 cm in four directions (east, west, south, and north) around the trunk base, which ensured that the main roots were not cut off. After the roots had been exposed, the fibrous roots with diameters of less than 2 mm were cut out and gently shaken to remove the soil attached to the root system. This soil was collected and used for the physical and chemical determination of soil properties. The roots and remaining attached soil, approximately a 1-mm layer, were placed into sterile sealed bags. A sterile scalpel was used to gently scrape the surface of the bark of the same tree at a height of 1.3 m and expose an area of approximately 200 mm in length and 100 mm in width. After being exposed, a phloem sample with a thickness of about 1 mm was removed and placed in a plastic bag. All samples were transported in a dry-ice environment.

### Processing of Samples

A sterilized soft brush was used to gently brush the rhizosphere soil off the root surface. The hairy roots in the rhizosphere soil were removed, then passed through a 1-mm sterile filter. The roots were cleaned eight times with sterile water and then placed in 15 mL phosphate-buffered saline (PBS) solution. An ultrasonic wave was applied twice at 50–60 Hz for 30 s. Water on the surface of the roots was absorbed with sterile absorbent paper. The phloem sample was rinsed twice with sterile water and excess water was absorbed. Samples were labeled S, R, and P, representing rhizosphere soil, roots, and phloem, respectively. The numbers 6, 8, and 10 were used to indicate the sampling months, corresponding to June, August, and October, respectively. The abbreviations A1, B1, and CK (for control) represented the line numbers, and the numbers 1–3 represented each of the three trees in each line (Supplementary Table 1). For example, S6-CK-1 would represent one sample of the June control line from rhizosphere soil. A total of 81 samples were collected in 3 months (27 samples per month), and samples were transported in a dry-ice environment.

### Extraction of DNA and Illumina MiSeq Sequencing

For DNA extraction from soil and plant tissue, HiPure Soil DNA and HiPure Stool DNA kits (Magen, Guangzhou, China) were used, respectively, according to the manufacturers’ protocols. The 16S rDNA target region of the ribosomal RNA gene and the internal transcribed spacer (ITS) target region were amplified via PCR, as follows: 95°C for 5 min, followed by 30 cycles at 95°C for 1 min, 60°C for 1 min, 72°C for 1 min, and a final extension at 72°C for 7 min. The primer sequences of the 16S rDNA V5–V7 region were 799F, AACMGGATTAGATACCCKG and 1193R, ACGTCATCCCCACCTTCC (Beckers et al., 2016); those of the fungal ITS2 rRNA region were ITS3_KYO2, GATGAAGAACGYAGYRAA and ITS4, TCCTCCGCTTATTGATATGC (Toju et al., 2012). The PCR reactions were performed in triplicate using a 50-μL mixture containing 10 μL 5 × Q5@ reaction buffer, 10 μL 5 × Q5@ high GC enhancer, 1.5 μL 2.5 mM dNTPs, 1.5 μL each primer (10 μM), 0.2 μL Q5@ high-fidelity DNA polymerase, and 50 ng template DNA. The PCR reagents were obtained from New England Biolabs (Ipswich, MA, United States). Amplicons were extracted from 2% agarose gels and purified using an AxyPrep DNA Gel Extraction Kit (Axygen Biosciences, Union City, CA, United States) according to the manufacturer’s instructions and quantified using the ABI StepOnePlus Real-Time PCR System (Life Technologies, Foster City, CA, United States). Purified amplicons were pooled in equimolar concentrations and paired-end sequenced (PE250) on an Illumina platform according to the standard protocols. The raw reads were deposited in the National Center for Biotechnology Information (NCBI) Sequence Read Archive (SRA) database (Accession Number: SAR17186426 to SAR17186587).

After the sequencing data had been obtained, we first performed quality-control processing to get high-quality clean tags. The clean tags were clustered into operational taxonomic units (OTUs) of ≥97% similarity using UPARSE (Edgar, 2013) (version 9.2.64) pipeline. The 16S rRNA and ITS representative OTU sequences were classified into organisms with a naive Bayesian model using the Ribosomal Database Project (RDP) classifier (Pruesse et al., 2007) (version 2.2) based on the SILVA (version 132) and ITS2 (version update_2015) databases (Ankenbrand et al., 2015), respectively, with a confidence threshold value of 0.8 (Wang et al., 2007). Based on the OTU sequence species annotation and abundance information, we carried out a follow-up biological information analysis.

### Soil Description and Analysis

Impurities in the soil were removed, and then the soil was passed through a 1-mm-mesh screen. The physical and chemical properties of the soil were measured as follows: soil pH was measured in a 1:2.5 (w/v) soil to water suspension using a pH meter according to the standard of NY/T1121.6-2006, organic matter (OM) using the potassium dichromate volumetric method according to the standard of NY/T 1121.6-2006, available nitrogen (AN) was measured with the alkaline hydrolysis diffusion method according to the standard of LY/T 1228-2015, available phosphorus (AP) was measured with molybdenum blue colorimetry according to the standard of NY/T 1121.7-2014, available potassium (AK) was measured with an ammonium acetate method using a flame photometer according to the standard of, NY/T889-2004, electrical conductivity (EC) using the electrode method according to the standard of HJ802-2016, and total salt (TS) content using the mass method according to the standard of van Reeuwijk (2006).

### Statistical Analysis

The Shannon diversity and Pielou’s evenness indexes were calculated using QIIME (Caporaso et al., 2010) (version 1.9.1). Sequence alignment was performed using Muscle (Edgar, 2004) (version 3.8.31), and a phylogenetic tree was constructed using FastTree (Price et al., 2010) (version 2.1). Then an unweighted UniFrac distance matrix was generated using the GuniFrac package (Lozupone and Knight, 2005) (version 1.0) in R project. Hierarchical clustering was performed using the R project in the Vegan package. Based on OTUs and species-abundance tables, the R language in the Vegan package was used (Oksanen et al., 2011) (version 2.5.3) to calculate the Bray–Curtis distance matrix for a principal coordinates analysis (PCoA), and the Spearman_approx distance was used for an analysis of similarities (ANOSIM) (Oksanen et al., 2011). A canonical correspondence analysis (CCA) was carried out using the R language in the Vegan package (Oksanen et al., 2011) (version 2.5.3). The Pearson correlation coefficients between environmental factors and species were determined using the R language psych package (Revelle, 2013) (version 1.8.4). Cytoscape software was used to generate a correlation network diagram. The functional group (guild) of fungi was inferred using FUNGuild (Nguyen et al., 2016) (version 1.0). The soil physical and chemical parameters and the abundance of microorganism functional groups were evaluated using IBM SPSS Statistics 26.0 software for statistical analysis, with significance set at the *P* < 0.05 level. Relative abundance comparison between groups was calculated using Welch’s *t*-test and the Wilcoxon rank test in the R project Vegan package (Oksanen et al., 2011) (version 2.5.3). Tukey’s honestly significant difference (HSD) and Kruskal–Wallis *H* tests were used to analyze relative abundance differences among multiple groups using R language in the Vegan package (Oksanen et al., 2011) (version 2.5.3).

## Results

### Microbial Community Richness and Diversity Patterns

**FIGURE 1:**
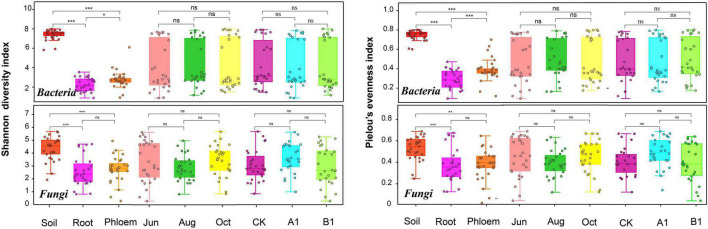
Shannon’s diversity and Pielou’s evenness of both the bacterial and fungal communities across the three habitat classifications (soil, roots, and stems), seasons (June, August, and October) and genotypes (CK, A1, and B1). For both bacteria and fungi, diversity differed significantly across habitats. *P < 0.05 between groups. **P < 0.01 between groups. ***P < 0.001 between groups. ns: There were no significant differences between groups.

The average numbers of effective tags of bacteria obtained from soil, root, and phloem samples in the amplicon were 112,670, 111,296, and 110,858, respectively. The average numbers of effective tags of fungi were 123,605, 108,658, and 99,144, respectively. The average lengths of the effective tags of bacteria and fungi were about 413.1 and 341 bp, respectively (Supplementary Table 2). Most of the reads could be classified, with a small proportion of unclassified reads (0–1.25% for bacteria and 0–0.2% for fungi). The numbers of OTUs in rhizosphere soil were saturated at about 1,800 (bacteria) and 260 (fungi), and the root and phloem samples were saturated at about 200 (bacteria) and 100 (fungi). According to a community α-diversity analysis (Figure 1), the diversity and evenness of the bacterial and fungal communities were significantly higher in rhizosphere soil than in roots and phloem, and the values were higher in phloem than in roots. There were no significant differences between the 3 months studied in terms of community diversity or evenness, and there were no significant differences between the transgenic lines and the control.

**FIGURE 2:**
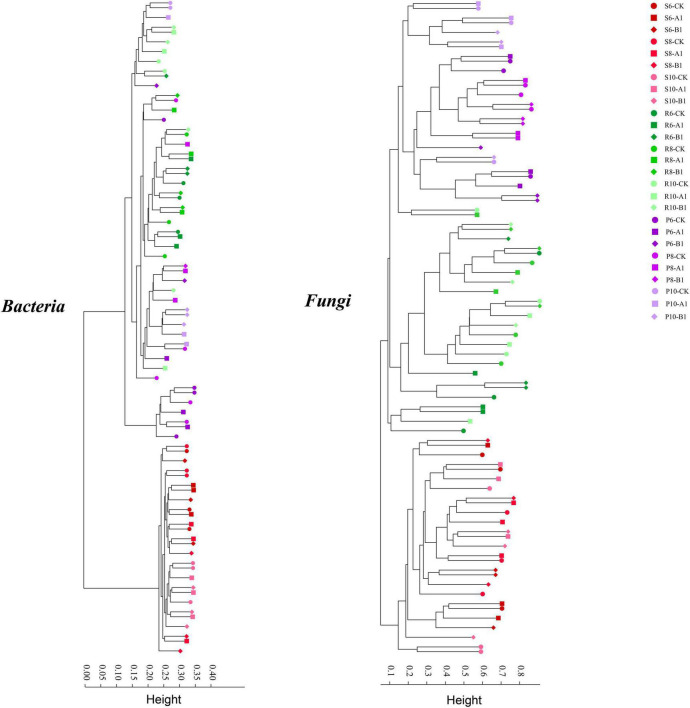
Results of a cluster analysis based on the UniFrac distance of all samples at a 3% cut-off OTU level in 16S and ITS.

**FIGURE 3:**
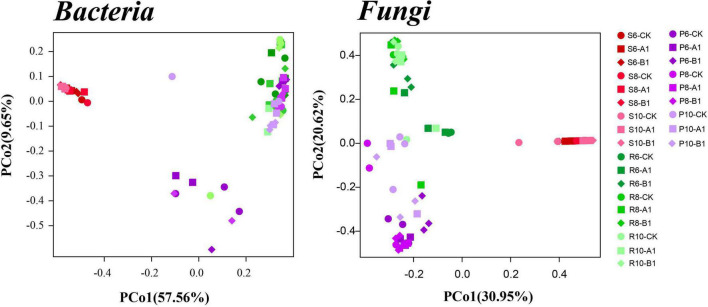
Results of a PCoA analysis of both the bacterial and fungal communities across the three habitat classifications (soil, roots, and stems), seasons (June, August, and October), and genotypes (CK, A1, and B1). Based on multivariate ANOVA results, habitat and season had greater impacts on bacterial and fungal community composition than did genotype (Supplementary Table 3).

A cluster analysis of the OTUs according to the unweighted_UniFrac showed that bacteria and fungi had the same pattern (Figure 2), that is, rhizosphere soil was clustered separately, and plant tissues (phloem and roots) were clustered into one cluster. The PCoA was used to evaluate the similarity of the microbial community structures (Figure 3). The first two principal components explained 66% of the bacterial community, and the rhizosphere soil community differed significantly from those of roots and phloem, which were too similar to distinguish between them. The first two principal components explained 50% of the fungal community, and the three habitats were clearly distinguished. For both bacteria and fungi, there was no clustering between the different months or between different lines. To support the results of the clustering and PCoA analyses statistically, analysis of similarity (ANOSIM), based on the Spearman_approx distance algorithm, was performed. The ANOSIM analysis results (Supplementary Table 3) showed that there were significant differences among the three habitats, and there were significant differences among seasons within each habitat (*P* < 0.05), but there were no significant differences between the transgenic lines and the control within each habitat.

### Site Chemical Characteristics

The environment is an important factor affecting the soil microbial community. The OM content, AN, TS, EC, and pH values in the rhizosphere soil of *Populus euphratica* varied significantly throughout the growing season (June vs. August vs. October), while the AP and AK were relatively stable (Supplementary Table 4). Throughout the site, there were no significant differences in the physical and chemical indexes between the rhizospheres of the transgenic and control poplar, indicating that the overall management conditions of the experimental forest were consistent.

**FIGURE 4:**
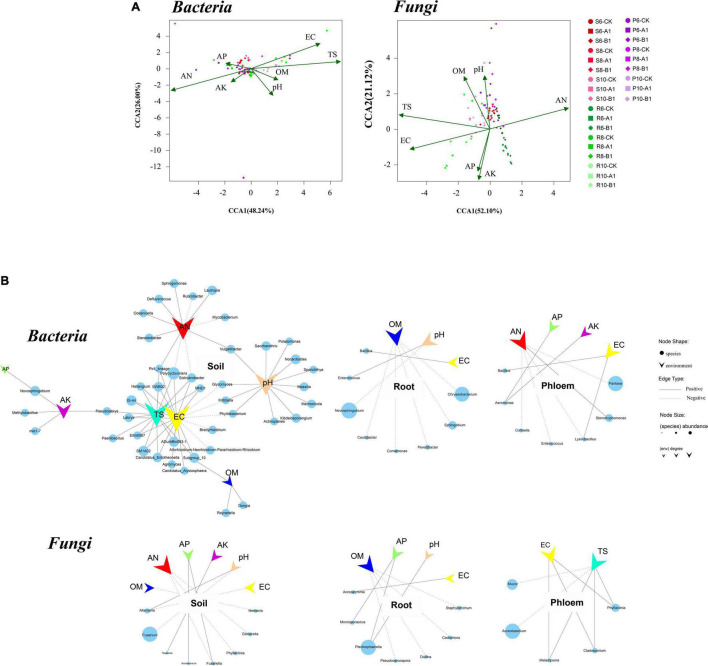
Canonical correspondence analysis (CCA) and correlation analysis between microbes and environmental factors. **(A)** Distance-based CCA of bacterial and fungal communities in all samples. **(B)** Correlations between genera and environmental factors. Each circle represents one genus of bacteria or fungi, and its size represents the average abundance across the corresponding habitats. The vee represents environmental factors. The figure shows only genera significantly associated with environmental factors, with a coefficient greater than 0.5.

The correlations between microbial community structure and environmental factors were calculated to examine the environmental factors that could lead to variation in microbial diversity. Overall, the physical and chemical properties had a greater impact on bacterial communities than on fungi, and on rhizosphere soils than on roots and phloem. AN, EC, and TS were the most important factors affecting bacterial and fungal communities (Figure 4A). In the bacterial community, the EC and TS indexes were positively correlated with nine phyla (*r* > 0.5, *p* < 0.05), including Acidobacteria, Verrucomicrobia, Planctomycetes, and Entotheonellaeota; the pH value was positively correlated with Actinomycetes; and the AN content was positively correlated with Planctomycetes at the genus level. The EC, TS, pH value, and AN content also had large effects. Regarding the endogenous tissue of plants, root bacteria were affected by the OM, pH, and EC, with the OM content having the largest effect, including on the highly abundant *Chryseobacterium*. Phloem bacteria were affected mainly by AN, AP, AK, and EC, with AN being the most important factor. In the fungal community, rhizosphere soil fungi were affected by AN, OM, AP, AK, pH, and EC, with AN and pH being the main factors, and pH specifically inhibiting the highly abundant *Fusarium* (Figure 4B). Root fungi were affected mainly by OM, AP, EC, and pH content in plant endogenous tissues, with AP and pH positively correlated with the abundant fungus *Plectosphaerella*. Phloem fungi were affected mainly by the EC and TS, with TS specifically inhibiting the highly abundant *Aureobasidium.*

### Microbial Community Composition

**FIGURE 5:**
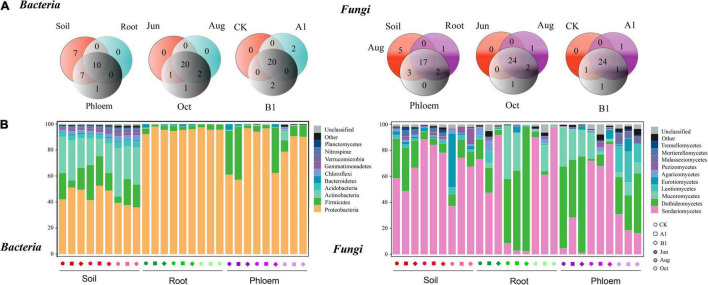
Bacterial and fungal community composition analysis. **(A)** Phyla/class distribution for both bacterial and fungal communities across the three habitat classifications (soil, roots, and stems), seasons (June, August, and October), and genotypes (CK, A1, and B1). **(B)** Average relative abundance (%) of bacterial phyla and fungi classes in soil, roots, and phloem for different seasons in CK, A1, and B1. The total relative abundances and significant effects of all phyla of bacteria and classes of fungi are listed in Supplementary Tables 4, 5.

As shown in Figure 5A, bacterial communities were annotated to 24 phyla, of which 24, 10, and 17 were annotated to rhizosphere soil, roots, and phloem, respectively. Nine phyla were common to all three habitats and seven appeared only in soil samples. The rhizosphere was dominated by Proteobacteria, Acidobacteria, and Firmicutes; the roots were dominated by Proteobacteria and Firmicutes; and phloem was dominated by Proteobacteria and Firmicutes (Figure 5B). There were significant differences in the abundances of a large number of phyla among habitats (Tukey’s HSD: *P* < 0.05, Supplementary Table 5). For example, the relative abundance of Proteobacteria was significantly lower in the rhizosphere than in roots and phloem (rhizosphere: 44%, root: 95%, and phloem: 81%). Actinobacteria (rhizosphere: 25.34%, root: 0.17%, and phloem: 1.14%) and Acinetobacter (rhizosphere: 3.96%, root: 0.01%, and phloem: 0.14%) were more abundant in soil than in roots and phloem. Firmicutes were less abundant in root samples (rhizosphere: 16.56%, root: 3.35%, and phloem: 16.56%) and Bacteroidetes were most abundant in phloem (rhizosphere: 0.83%, root: 16.56%, and phloem: 1.04%). Fungal community was annotated to 28 classes, of which 25, 20, and 22 were annotated to rhizosphere soil, root, and phloem, respectively. Seventeen were common to the three habitats, and five classes appeared only in rhizosphere soils. One class appeared only in roots. Sordariomycetes and Dothideomycetes dominated the rhizosphere soil, and Sordariomycetes, Dothideomycetes, and Mucoromycetes dominated the root and phloem. The abundances of a large number of classes differed significantly among different habitats (Tukey’s HSD: *P* < 0.05, Supplementary Table 5). For example, the relative abundance of Sordariomycetes was significantly higher in rhizosphere soil and roots than in phloem (soil: 67.04%, root: 76.21%, and phloem: 12.63%). The relative abundance of Dothideomycetes was highest in phloem and lowest in roots (rhizosphere: 15.23%, root: 6.55%, and phloem: 54.49%). The relative abundance of Mucoromycetes was highest in phloem, and it was absent from rhizosphere soil (rhizosphere: 0.00%, root: 5.49%, and phloem: 18.49%).

In the comparison between different seasons (Figure 5A), 20 phyla were common between June, August, and October in the bacterial community, One phyla appearing only in October. In the rhizosphere soil, there were significant seasonal changes in 11 phyla (Figure 5B and Supplementary Table 6). The abundance of Proteobacteria was significantly higher in June and August than in October (June: 47.45%, August: 47.57%, and October: 37.58%). Actinobacteria had the lowest abundance in August (June: 27.13%, August: 18.71%, and October: 30.19%), while Acidobacteria had the highest abundance in October (June: 3.36%, August: 3.44%, and October: 5.06%). The abundance of Bacteroidetes in roots (June: 1.21%, August: 1.28%, and October: 0.02%) and phloem (June: 2.09%, August: 0.94%, and October: 0.10%) was significantly lower in October than in June and August. There were 24 classes that were common between June, August, and October in the fungal community, with Umbelopsidomycetes appearing only in August and Paraglomeromycetes appearing only in October. In the rhizosphere soil, eight classes differed significantly among seasons. The abundance of Sordariomycetes was significantly lower in August than in June and October (June: 57.95%, August: 83.58%, and October: 59.58%), and that of Dothideomycetes was significantly higher in June than in August and October (June: 28.29%, August: 5.90%, and October: 11.49%). The abundance of Eurotiomycetes was significantly higher in October than in June and August (June: 1.97%, August: 1.34%, and October: 15.08%). There were no significant seasonal changes in the abundances of classes in the fungal community in roots. The abundances of classes in phloem fluctuated seasonally, with 16 classes showing significant differences. For example, the abundance of Bacteroidetes gradually decreased from June to October (June: 2.10%, August: 0.94%, and October: 0.10%), while those of Dothideomycetes (June: 69.03%, August: 60.56%, and October: 33.88%) and Mucoromycetes (June: 23.78%, August: 24.38%, and October: 7.33%) decreased significantly in October, and those of Sordariomycetes (June: 4.58%, August: 11.42%, and October: 21.89%), Leotiomycetes (June: 0.04%, August: 0.19%, and October: 19.34%), and Malasseziomycetes (June: 0.35%, August: 0.72%, and October: 6.27%) gradually increased from June to October.

In the bacterial community, 20 phyla were common among the A1, B1, and CK lines (Figure 5A), with two endemic phyla (Deinococcus-Thermus and Fibrobacteres) in controls and two endemic phyla (Omnitrophicaeota and Thaumarchaeota) in B1. Compared to CK, there were no significant differences among the phyla in the three habitats (Welch’s *t*: *P* > 0.05). Regarding the fungal community, 24 classes were common among A1, B1, and CK, with one endemic class (Umbelopsidomycetes) in A1, and one (Paraglomeromycetes) in B1. When A1 and B1 were compared to CK, there were no significant differences in abundance identified for any classes (Welch’s *t*: *P* > 0.05) (Supplementary Table 7).

### Identification of Shared Operational Taxonomic Units and Indicator Species Analysis

**FIGURE 6:**
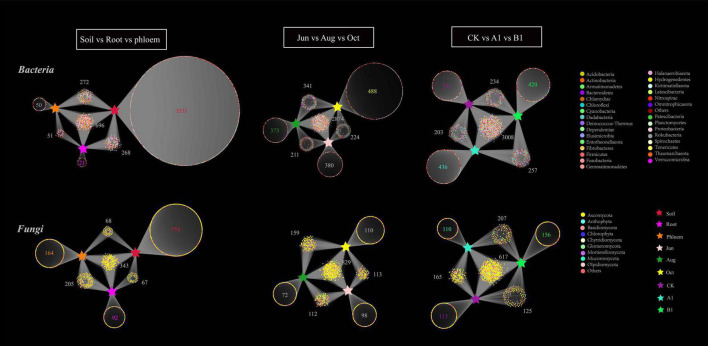
Bipartite association network of both bacterial and fungal communities across the three habitat classifications (soil, roots, and stems), seasons (June, August, and October), and genotypes (CK, A1, and B1). Each circle corresponds to an OTU, and its color represents the OTU annotation of bacteria at the phylum level and fungi at the class level. The stars represent different groups.

A bipartite association network revealed the distribution and annotation of OTUs in the different groups. As can be seen in Figure 6, there were 4,891 bacterial OTUs enriched in all of the samples, mainly in the Proteobacteria. The OTUs of rhizosphere soil, root, and phloem were 4,767, 1,036, and 1,069, respectively. In bacterial community, there were 696 (shared 14%) common OTUs among rhizosphere soil, root, and phloem, and each of the habitats having 3,531, 23, and 50 unique OTUs, respectively; the numbers of significantly enriched OTUs were 450, 33, and 61, respectively (Supplementary Table 8). The OTUs of June, August, and October were 3,689, 3,799, and 3,927, respectively. By month, there were slightly more OTUs of bacteria in October than in June and August. There were 2,874 OTUs (59%) shared among June, August, and October, with 380, 373, and 488 unique bacterial OTUs, respectively. The numbers of significantly enriched OTUs were 127, 101, and 204, respectively (Supplementary Table 9). The OTUs of CK, A1, and B1 lines were 3,778, 3,904, and 3,919, respectively. There were 3,211 bacteria OTUs (66%) shared between the A1 and CK lines, with 693 and 567 unique OTUs, respectively. There were 3,241 bacteria OTUs (66%) shared between the B1 and CK lines, with 677 and 536 unique OTUs, respectively. Several OTUs in the A1, B1, and CK lines were significantly enriched (*q* > 0.05) (Supplementary Table 10).

**FIGURE 7:**
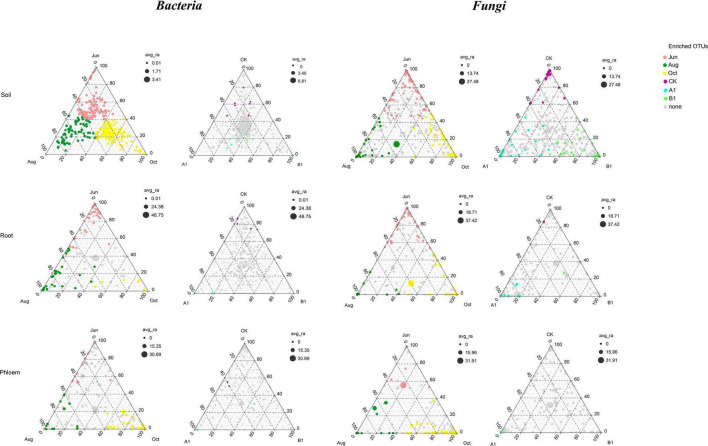
Ternary plot depicting the relative abundances of all OTUs for bacterial and fungal communities across the three habitat classifications (soil, roots, and stems), seasons (June, August, and October), and genotypes (CK, A1, and B1). Each point corresponds to an OTU, its position represents its relative abundance with respect to each group, and its size represents the average across all three groups. Colored circles represent OTUs enriched in one group compared to the others.

In total, 1,493 fungal OTUs were enriched in all of the samples (Figure 6), with most of them in the Ascomycota. The OTUs of rhizosphere soil, root, and phloem were 1,032, 707, and 780, respectively. There were 343 common OTUs (shared 23%) among rhizosphere soil, roots, and phloem, and each of the habitats having 554, 92, and 164 unique OTUs; 93, 39, and 45 were significantly enriched, respectively (Figure 7 and Supplementary Table 8). The OTUs of June, August, and October were 1,152, 1,172, and 1,211, respectively. By month, 829 OTUs (56%) were shared among June, August, and October, with 72, 98, and 110 unique OTUs; 109, 54, and 158 were significantly enriched, respectively (Supplementary Table 9). The OTUs of CK, A1, and B1 lines were 1,020, 1,099, and 1,114, respectively. There were 782 fungal OTUs (52%) shared between the A1 and CK lines, with 317 and 228 unique OTUs, respectively. There were 742 fungal OTUs (50%) shared between the B1 and CK lines, with 361 and 278 unique OTUs, respectively. There were no significantly enriched OTUs in the A1, B1, and CK lines (*q* > 0.05). Four fungal OTUs were significantly enriched in the CK lines, two OTUs were significantly enriched in the B1 lines (*q* > 0.05), and no OTUs were significantly enriched in the A1 lines (Figure 7 and Supplementary Table 10).

### Distribution of Functional Groups

**FIGURE 8:**
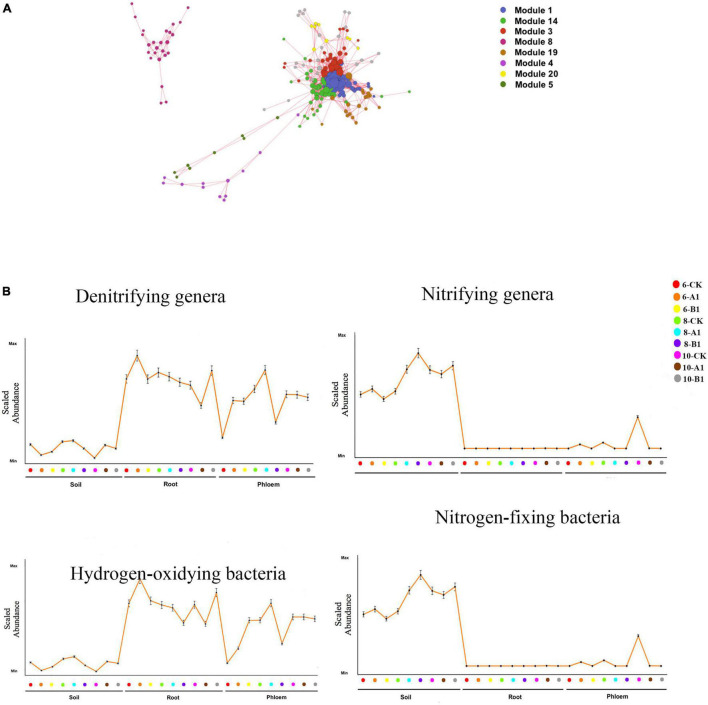
Functional analysis of the bacterial communities. **(A)** OTU co-abundance network revealing the modules of OTUs associated with functional bacteria. Only uniquely associated OTUs or those associated with all four functional genera and with significant associations (i.e., correlation greater than 0.5) are shown. Each node represents an OTU, and each module is displayed in a different color. **(B)** Mean abundance profile for the OTUs in the functional bacteria groups across different samples. The position along the *x*-axis corresponds to different samples. Error bars represent the SE. The *x*- and *y*-axes have no scale.

Interpreting the biological implications of the diversity patterns revealed by NGS studies requires knowledge of the key functional attributes of different microbial species. Based on OTU abundance, a Pearson correlation was calculated between each OTU for the four functional bacteria groups (nitrifying genera, denitrifying genera, hydrogen-oxidizing bacteria, and nitrogen-fixing bacteria), and eight OTU modules (Figure 8A and Supplementary Table 11) significantly correlated with the four functional bacteria (*R* > 0.5, *P* < 0.05) were screened. The dominant nitrifying genera included *Nitrosospira*, *Nitrospira*, *Nitrobacter*, *Nitrosomonas*, *Candidatus_Nitrocosmicus*, *Nitrolancea*, and *Nitrospira*. The abundance of nitrifying bacteria was significantly greater in rhizosphere soil than in roots and phloem (Figure 8B and Supplementary Table 12). Within the bacterial community, 16 denitrifying bacteria were identified, among which *Paenibacillus* and *Bacillus* were dominant. *Paenibacillus* was dominant in endogenous tissue, and *Bacillus* was dominant in rhizosphere soil (Supplementary Table 13). The abundance of denitrifying bacteria was significantly greater in root and phloem than in rhizosphere soil (Figure 8B and Supplementary Table 12). The abundance of nitrogen-fixing microorganisms can reflect the nitrogen-fixing potential. The nitrogen-fixing bacteria observed in this study included *Azospirillum*, *Bradyrhizobium*, *Rhizobium*, *Mesorhizobium*, and the *Allorhizobium-neorhizobium-pararhizobium-rhizobium* complex; abundance was highest for this latter complex (Supplementary Table 13). The highest abundance of nitrogen-fixing bacteria was observed in rhizosphere soil, followed by the root system, with almost no nitrogen-fixing bacteria in the phloem. Moreover, Pseudomonas was observed to be dominant (Supplementary Table 13). The abundance of hydrogen-oxidizing bacteria was greater in the root system than in the phloem, and was lowest in rhizosphere soil. By season, only the abundance of nitrogen-fixing bacteria showed obvious seasonal changes, being significantly greater in June and August than in October (Figure 8B and Supplementary Table 12). There were no significant differences in the abundance of functional bacteria between the transgenic lines and the control (Figure 8B and Supplementary Table 12).

**FIGURE 9:**
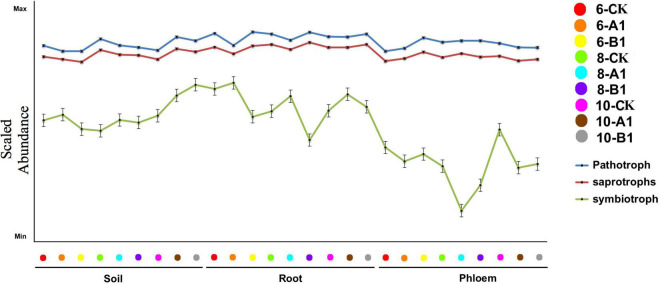
Abundance of trophic groups in the fungal community across different samples. The vertical represents the Log10 transformation of the sample stages.

Generally, fungi species are grouped into saprotrophs, symbiotrophs, and pathogens, and then each of these groups is divided into functional guilds of species that have different growth strategies and nutrient-acquisition capabilities (Peay et al., 2016). Throughout all of the habitats studied, pathogens were the most abundant trophic group, followed by saprotrophs, with symbiotrophs having the lowest abundance (Figure 9). The abundance of pathogens and saprotrophs was significantly greater in roots than in rhizosphere soil and phloem, while symbiotrophs were mainly present in rhizosphere soil and roots (Supplementary Table 14A). The abundance of most guilds varied greatly among different habitats (Supplementary Table 14B). There were more fungal functional guilds in roots than in soil and phloem. There were no obvious differences in the abundance of trophic groups and fungal guilds in different seasons or among the different lines.

## Discussion

The widespread application of *Bacillus thuringiensis* (Bt) insecticidal protein genes via plant genetic transformation has alleviated the harm caused by insects to the agriculture and forestry industries, and the release of pesticides into the environment has been reduced (Wang et al., 2018). Assessing and monitoring the ecological safety of genetic-transformation technology is necessary for the promotion and application of genetically modified plants (Filion, 2008). Microorganisms are very important to the growth and development of plants, but their colonization mechanism in plants is still unclear. Exploring the changes in microbial communities in different ecological parts of *PEN*, the main poplar species planted in the North China Plain, could make complex microbial communities better able to serve agriculture and ecology. In this study, abundant bacterial and fungal OTUs in environmental samples were detected, and the number of bacterial OTUs was larger than that of fungi in the rhizosphere soil (bacterial OTUs: 1,944, fungal OTUs: 299), root system (bacterial OTUs: 242, fungal OTUs: 122), and phloem (bacterial OTUs: 182, fungal OTUs: 102). Previous studies have shown that the diversity and species richness of bacteria and fungi in individual communities vary with habitat, but the species richness of bacteria is usually higher than that of fungi (Hinchliff et al., 2015); our results are consistent with this. In this study, the number of OTUs annotated by sequencing was relatively high compared to previous studies (Bue et al., 2009; Beckers et al., 2017), and a higher sequencing depth was beneficial to fully characterize the microbial community.

### Effects of Gene Transformation on the Microbial Community

Bt has insecticidal action, which may affect the soil microflora, particularly some functional bacteria, and reduce their numbers (Motavalli et al., 2004). For example, the endophytic fungal community of transgenic corn leaves has been changed (da Silva et al., 2016), the diversity of the endophytic microbial community in the root system of genetically modified oilseed rape is lower than that of non-transgenic oilseed rape (Dunfield and Germida, 2001; Siciliano and Germida, 2010), and overexpression of malate dehydrogenase in alfalfa affects the diversity of rhizosphere bacteria and the availability of soil nutrients (Tesfaye et al., 2003). However, most studies have shown that transgenic plants have no influence on the rhizosphere or endophytic microbial communities (Andreote et al., 2008; Cotta et al., 2014; Nimusiima et al., 2015; Timm et al., 2016). In genetically modified poplars, some studies have shown that overexpression of tannin in *Populus tremula* × *tremuloides* has a significant impact on the fungal and bacterial communities of rhizosphere soil (Winder et al., 2013), while other studies have not found that transgenic poplar has an impact on microbial communities (Oliver et al., 2008; Danielsen et al., 2012; Zuo et al., 2018; Wang et al., 2019). In this study, transgenic *PEN* with Cry1Ac-Cry3A-BADH and Cry1Ac-Cry3A-NTHK1 genes had the same richness, uniformity, and diversity as non-transgenic poplars for both bacteria and fungi communities. The transgenic and non-transgenic lines cannot be distinguished by PCoA or cluster analysis, and no statistical differences were found in bacterial phyla and fungi classes between them, indicating the community structure and composition of the transgenic lines and control were similar. This result is consistent with the previous study (Wang et al., 2019). It was compared the OTUs of transgenic poplar to those of a control and found that 49.63 and 37.63% of the OTUs were shared with bacteria and fungi, respectively. In our study, >66% of the bacterial OTUs and >50% of the fungal OTUs were shared between transgenic *PEN* and non-transgenic *PEN.* This confirmed that the percentage of shared OTUs was relatively high.

In this study, small numbers of low-abundance OTUs were significantly enriched in the A1 and CK lines. Even in the same area, there are differences in the microorganisms present due to soil heterogeneity (Brockett et al., 2012) and the presence of different kinds of plants. Even individuals of the same plant species growing in the same environment have unique endophytic bacterial communities (Watt et al., 2006; Gaiero et al., 2013). The fungal community had more specific OTUs than the bacterial community, consistent with previous research (Wang et al., 2019). In terms of the historical contingency, the final composition of a community is determined by the order of arrival of each species (Peay et al., 2016). Both bacteria and fungi had a few specific OTUs, but no difference was observed between transgenic lines and the control in terms of community function. On the other hand, microorganisms can be used as diagnostic groups to indicate environmental conditions, plant health, and soil fertility (Yu et al., 2012).

If low-abundance enriched species at the OTU level in transgenic fungi are identified as sensitive groups, attention should be given to them because they may reflect specific health conditions of soil or endogenous tissues (Dohrmann et al., 2013). In summary, the transgenic event did not affect the rhizosphere soil or the endogenous bacterial and fungal communities of *PEN.* Because our study focused on transgenic poplar planted for 4 years, it will be necessary to monitor the microbial community continuously during the whole growth cycle of these transgenic poplars. In addition, more comprehensive sequencing technology, such as metagenomic sequencing technologies can be applied to microbial community research of transgenic plants.

### Microbial Community Structure Across Habitats

Microbes show strong environmental selection, and the microbial communities in plant roots differ from those in the rhizosphere. We observed significant differences in microbial communities in different ecological regions of *PEN*. The richness and diversity of soil bacterial and fungal communities were significantly higher in rhizosphere soil than in roots and phloem, and the differences between the root and phloem communities were relatively small. The same niche differentiation has been reported in poplar, cactus, and willow (Fonseca-García et al., 2016; Tardif et al., 2016; Beckers et al., 2017; Cregger et al., 2018). Different plant tissues can contain different endophytic communities (Cregger et al., 2018). Johnston-Monje and Raizada labeled endophytes with green fluorescent protein (GFP) and found that endophytes can be transported from seeds to the roots and stems of plants; in addition, endophytes injected into stems entered the roots and rhizosphere, indicating that there may be continuous movement of organisms throughout the whole root microbial community (Johnston-Monje and Raizada, 2011). There is an abundant microbial population in the soil, with some bacteria able to actively or passively travel through the endodermis and pericycle to the xylem vessels and eventually colonize the plant (Hardoim et al., 2008). Most endophytes that promote plant growth belong to this category, and it is speculated that the endophytic microorganisms in the root and phloem of *PEN* also have certain communicative activities, and therefore the microbial communities were similar. In this study, the factors controlling variation in bacterial and fungal communities differed in different tissues. In bacterial communities, diversity and evenness significantly differed in roots and phloem, while there were no significant differences in fungal communities. This may be related to the community characteristics of bacteria and fungi. Fungi are more resistant to dryness, and there is usually a tendency for the same species to be present in different tissues of a host plant (Tardif et al., 2016). Previous studies have shown that the bacterial richness of transgenic poplar is higher in roots than in stems (Cregger et al., 2018); in our study, bacterial richness and diversity were greater in phloem than in roots, which may be related to the sampling density and sampling range of roots. The greater the sampling density, the higher the diversity of microorganisms. Because roots have various functions, there may be differences in the numbers of microorganisms present in the different root-diameter ranges of the poplar trees.

The bacterial community in rhizosphere soil was dominated by Proteobacteria, while Firmicutes and Actinobacteria were also abundant. Proteobacteria was also dominant in roots and phloem and is a common phylum in ecosystem sequencing surveys. The ratio of Proteobacteria to Acidobacteria is considered an index of the soil nutrition level (Hardoim et al., 2008; Castro et al., 2010). In this study, the ratio of Proteobacteria to Acidobacteria is 11.11. Eutrophic soil is conducive to the development of Proteobacteria, while nutrient-deficient soil is conducive to the growth of Acidobacteria. In the rhizosphere soil of *Trifolium repens* and *Lolium perenne*, the presence of Proteobacteria is considered related to eutrophication conditions (Marilley and Aragno, 1999). Many other factors may influence the ratio of Proteobacteria to Acidobacteria. For example, the relative abundance of Acidobacteria in soil has been proven to be related to soil pH (Castro et al., 2010) and relative humidity (Wang et al., 2007). Jin et al. (2014) identified Bacteroides in stems and leaves but not in root samples. Our results are similar with Yang et al. (2017) that Sclerenchyma and Bacteroides were more abundant in the endophytic community of leaves than in root samples. The abundances of Bacteroides and thick-walled fungi were higher in stems than in roots, which may be related to niche preference and may also be controlled by the plants themselves. Microorganisms perform a redox process through the global carbon and nitrogen cycle displaying consortium dynamics (McInerney et al., 2009), i.e., they cooperate in networks, supplying each other with critical nutrients for growth and survival. In this study, nitrogen-fixing bacteria were highly abundant in rhizosphere soil and roots, hydrogen-oxidizing bacteria and denitrification bacteria were present mainly in plant tissues, and nitrification bacteria were present mainly in rhizosphere soil, which may be related to environmental selection and plant requirements (Winder et al., 2013). We speculate that this is a healthy distribution pattern, and nitrogen is a limiting nutrient for plant growth in many soil ecosystems. Nitrifying bacteria in the soil can easily form nitrate, which becomes a nutrient substance that can be absorbed and utilized by plants, which is conducive to the normal growth of organisms. Denitrification causes nitrogen loss and produces nitrous oxide, which is a greenhouse gas. In this study, the low number of denitrifying bacteria in rhizosphere soil was beneficial to maintaining the AN for plants and reducing the negative impact on the environment (Philippot et al., 2007). As a kind of growth-promoting bacteria in the rhizosphere, hydroxide bacteria can synthesize indoleacetic acid, which is adsorbed on the surface of roots and then utilized by plants. At the same time, it can stimulate cell proliferation and growth (Winder et al., 2013) together with endogenous indoleacetic acid in plants. The greater number of hydrogen-oxidizing bacteria in the endogenous tissue of *Populus deltoides* may enhance its own growth and development.

The dominant fungi were Sordariomycetes, Dothideomycetes, and Mucoromycetes. Mucoromycetes were present mainly in endophytic tissues (Wanasinghe et al., 2018). Sordariomycetes is a class of ubiquitous fungi worldwide found in almost all ecosystems and includes pathogens, plant endophytes, animal pathogens, and fungal parasites (Maharachchikumbura et al., 2016). A previous study reported that the dominant fungi in the stem of poplar is Dothideomycetes, while there are no Mucoromycetes (Wang et al., 2019). There has been little research on the role of Mucoromycetes in plants. Mucoromycetes and Glomeromycetes are homologous strains (Rimington et al., 2015), while Glomeromycetes can form mycorrhizae in most plants. One study showed that Mucoromycetes have a symbiotic relationship with lower terrestrial plants, such as liverworts, hornworts, and lycopods (Lin et al., 2014). With functional information, it can be predicted how a fungal population will respond to changes in the environment. Among most higher plants, poplar is unusual in that it can combine with the endomycorrhizae of Ballycotina but also coexist with the ectomycorrhizae of Ascomycotina and Basidiomycorrhiza. These mycorrhizal fungi (including arbuscular mycorrhizae and ectomycorrhizae) can easily colonize the roots of poplar (Vozzo and Hacskayl, 1974; Baum and Makeschin, 2015). However, less than 2% of the fungi in this study were classified as mycorrhizae, and most were pathogenic and saprophytic fungi. There were many reasons for the low number of mycorrhizae. Some common chemicals in poplar can trigger the growth of fungal pathogens, while a large number of pathogens hinder mycorrhizal colonization (Cregger et al., 2018). On the other hand, large inputs of nitrogen (Peay et al., 2016), excessive levels of AP (Rozek et al., 2018), and saprophytic bacteria may also lead to a decrease in the abundance of ectomycorrhizal fungi.

### Influence of Environmental Factors on the Microbial Community

Various climatic factors and their interactions can change the characteristics and processes of ecosystems, thus directly or indirectly affecting microbial communities (Podolich et al., 2015; Whitaker et al., 2018). In this study, the bacterial community in the rhizosphere soil of *PEN* changed substantially between seasons, with significant changes in the abundances of Actinobacteria, Proteobacteria, and Acidobacteria, while endogenous bacteria were relatively stable, which may be caused by seasonal fluctuations in the physical and chemical properties of the soil. Rhizosphere microorganisms are in direct contact with the soil, and therefore the bacterial community is greatly affected by the soil’s physical and chemical properties (Yaish et al., 2016; Thiem et al., 2018). A study on transgenic *Brassica napus* L. in five field sites showed that there were differences in the endophytic bacterial communities among the different sites (Dunfield and Germida, 2001), and pH and soil OM content may have significant effects on the endophytic microbial community of poplar (Wang et al., 2019). One study found that pH was the best predictor of changes in soil bacterial communities, with a reported gradient change in the abundance of Acinetobacter and Actinomycetes (Hartman et al., 2008). A study on an 80-mile-long coniferous forest in a coastal zone with land uplift found a strong correlation between OM characteristics and the structure of the vegetation and microbial communities (Merilä et al., 2010). In our study, the pH value of rhizosphere soil was positively correlated with actinomycetes, salt content (TS, EC) was positively correlated with many phyla such as Acidobacteria, and AN also affected the abundance of multiple genera including *Sphingomonas* (White et al., 1996). A new microbial resource, *Sphingomonas*, is the main strain used to degrade aromatic pollutants, and it has broad application prospects in the field of environmental pollution control and biotechnology. In endogenous tissues, soil physicochemical properties affect relatively few bacterial genera, the root system is affected mainly by OM content, and phloem is affected by AN. Fungal communities are fundamentally different from bacterial communities and are usually more sensitive to the regional climate edaphic and spatial variables (Tedersoo et al., 2014; Peay et al., 2016). Available water is one of the most important environmental conditions that determine the diversity and composition of fungi. Adequate water content is very important to maintain cell expansion pressure, which is necessary for the expansion of hyphae and the diffusion and absorption of resources through cell membranes. Studies have shown that fungal richness is positively correlated with water-related climate variables (such as annual precipitation and potential evapotranspiration) (Pellissier et al., 2014). Available nutrient resources are also an important driving factor of fungal diversity. Compared to bacteria, fungi are more tolerant of acidic conditions and less sensitive to changes in pH values (Rousk et al., 2009). In this study, we found that OM content affected mainly rhizosphere endophytes, and pH values were negatively correlated with *Fusarium*. *Fusarium* is an important fungus that is widely distributed in plants and soil and can cause diseases in many commercial crops such as melon, pepper, potato, and tomato. It is also the cause of pink ear rot in rice and corn (Leslie et al., 2007). The salt content of soil affects mainly phloem endophytes, including *Aureobasidium pullulans* (Chi et al., 2009), an important yeast that can produce melanin in biotechnological applications. It has an inhibitory effect on *Aureobasidium* in phloem.

## Conclusion

A field experiment was conducted under the background of natural variation. Based on 16S rDNA and ITS amplicon sequencing, the responses of rhizosphere soil and endogenous microorganisms of *PEN* to transgenic events were studied. The results show that the diversity and evenness of the bacterial microbial community were significantly greater than those of the fungal community, and fungi had greater local environmental heterogeneity than did bacteria. Habitats and seasonal changes were the major driving factors of changes in microbial communities, and microbial community richness in rhizosphere soil, roots, and phloem of *PEN* was not affected by transgenic events.

## Data Availability Statement

The datasets presented in this study can be found in online repositories. The names of the repository/repositories and accession number(s) can be found below: National Center for Biotechnology Information (NCBI), Sequence Read Archive (SRA) database; PRJNA786956 (or SAR17186426–SAR17186587r).

## Author Contributions

MY designed the study. YH, YD, and YR collected and prepared samples for amplicon sequencing. YH, YD, YR, SW, and YL performed statistical analysis and visualization. YH, XL, and YD wrote the original draft. KD reviewed the manuscript. XL revised the manuscript. MY wrote, reviewed, and edited the manuscript. All authors read and approved the final manuscript.

## Conflict of Interest

The authors declare that the research was conducted in the absence of any commercial or financial relationships that could be construed as a potential conflict of interest.

## Publisher’s Note

All claims expressed in this article are solely those of the authors and do not necessarily represent those of their affiliated organizations, or those of the publisher, the editors and the reviewers. Any product that may be evaluated in this article, or claim that may be made by its manufacturer, is not guaranteed or endorsed by the publisher.
